# Electrocardiogram Quality Assessment with a Generalized Deep Learning Model Assisted by Conditional Generative Adversarial Networks

**DOI:** 10.3390/life11101013

**Published:** 2021-09-26

**Authors:** Xue Zhou, Xin Zhu, Keijiro Nakamura, Mahito Noro

**Affiliations:** 1 Biomedical Information Engineering Lab, The University of Aizu, Aizu-Wakamatsu, Fukushima 965-8580, Japan; d8212108@u-aizu.ac.jp; 2 Division of Cardiovascular Medicine, Toho University Ohashi Medical Center, Tokyo 153-8515, Japan; 3 Division of Cardiovascular Medicine, Odawara Cardiovascular Hospital, Tokyo 250-0873, Japan; noro@ojh.or.jp

**Keywords:** data augmentation, deep learning, ECG quality assessment

## Abstract

The electrocardiogram (ECG) is widely used for cardiovascular disease diagnosis and daily health monitoring. Before ECG analysis, ECG quality screening is an essential but time-consuming and experience-dependent work for technicians. An automatic ECG quality assessment method can reduce unnecessary time loss to help cardiologists perform diagnosis. This study aims to develop an automatic quality assessment system to search qualified ECGs for interpretation. The proposed system consists of data augmentation and quality assessment parts. For data augmentation, we train a conditional generative adversarial networks model to get an ECG segment generator, and thus to increase the number of training data. Then, we pre-train a deep quality assessment model based on a training dataset composed of real and generated ECG. Finally, we fine-tune the proposed model using real ECG and validate it on two different datasets composed of real ECG. The proposed system has a generalized performance on the two validation datasets. The model’s accuracy is 97.1% and 96.4%, respectively for the two datasets. The proposed method outperforms a shallow neural network model, and also a deep neural network models without being pre-trained by generated ECG. The proposed system demonstrates improved performance in the ECG quality assessment, and it has the potential to be an initial ECG quality screening tool in clinical practice.

## 1. Introduction

Electrocardiogram (ECG) is widely used for cardiovascular disease diagnosis, treatment, and daily personal health monitoring via wearable devices [1,2]. ECG signals are expected to have sufficient signal quality to extract temporal and morphological information for further analysis, such as heart rate variability (HRV) analysis and arrhythmia classification [3,4]. Low-quality ECG signals owing to baseline wander, muscle artifacts, and power-line interferences may cause false ECG arrhythmia alarms [5]. Additionally, ECG collected by wearable devices may include severe electrode motion artifacts, plain lines, and huge impulses due to lead-off. In particular, electrode motion artifacts may be treated as ectopic beats and cannot be removed by simple filters. This is one of the major factors that cause alarm fatigue [6,7,8]. In clinical practice, before disease diagnosis, low-quality ECG signals are expected to be removed through manual screening by technicians. However, manual quality screening is time-consuming, laborious, and experience-dependent. Therefore, a reliable automatic ECG signal quality assessment system is significant for ECG technicians and cardiologists.

To date, many studies have been conducted on ECG quality assessment. PhysioNet organized a challenge in cardiology in 2011 to classify 12-lead ECG signals as acceptable or unacceptable [6,9]. Quesnel et al. evaluated the quality of ECG signals contaminated with various levels of motion artifacts. They segmented PQRST complexes, which were aligned and averaged to form an estimate of true PQRST complexes. Then, a signal-to-noise ratio (SNR) was estimated by comparing each PQRST complex to the average PQRST complex. In this way, they got a 0.89 Pearson correlation coefficient between estimated and real SNRs [10]. The machine learning technique was also implemented in the ECG quality assessment. Redmond et al. used a Parzen window classifier to classify noisy and clean ECG, and got 82% and 78.7% accuracies using human and automatic annotation features, respectively [11]. Shahriari et al. obtained ECG signals from an ECG alarm study at the University of California, San Francisco (UCSF) and PhysioNet Computing in Cardiology Challenge 2011. They developed an image-based ECG quality assessment method. They computed a structural similarity measure (SSIM) at first, and then selected representative ECG images from the training dataset as templates. The SSIM between each ECG image and all the templates were used to build the features and input them into a linear discriminant analysis classifier. The classifier achieved 93.1% and 82.5% accuracies in the UCSF and Cardiology Challenge 2011 database, respectively [12]. Zhao et al. manually extracted six features, such as R peaks, the power spectrum distribution of QRS complexes, and so forth to build fuzzy vectors. They used the fuzzy comprehensive evaluation method as a feature analysis module. Their model demonstrated a 94.67% accuracy, 90.33% recall, and 93.00% specificity, training and testing on data from the PhysioNet computing in Cardiology Challenge 2011 and 2017 [13]. In 2019, Moeyersons et al. used data from a sleep study collected by the University Hospital Leuven, PhysioNet Computing in Cardiology Challenge 2017 and MIT-BIH Noise Stress Test Database with manual labels. They segmented the ECG signal into 5 s episodes after filtering. Each episode was characterized by an autocorrelation function, and then three features were extracted and fed to a RUSBoot classifier. For Challenge 2017 and Sleep Study Datasets, they obtained a recall of 79.4% and 96.6%, specificity of 78.7% and 84.8%, and area under the curve of 0.928 and 0.970, respectively [14]. More recently, Fu et al. assessed the quality of wearable ECG signals collected via Lenovo H3 Devices. They compared three machine learning algorithms: the support vector machine (SVM), least-squares SVM (LS-SVM), and long short-term memory (LSTM) with manually extracted features. The LSTM models achieved the best performance with 95.5% accuracy [15].

The above studies usually follow three procedures. The first procedure is signal prepossessing, such as filtering. Then, feature extraction is the most important step, and directly affects the model’s performance. However, no gold standard exists to identify necessary, effective, or redundant features. As a result, feature extraction usually depends on the experiences of researchers. The final step is model development using machine learning techniques or designing decision rules via setting thresholds or computing related statistical values based on extracted features. However, feature extraction, decision rules, or threshold-making are experience-dependent, and it is hard to cover or find out all significant features, such as QRS-related information [11,13], R-peak-related information [13], or autocorrelation function-related features [14]. In addition, significant features may vary with decision strategies and models. Furthermore, features manually extracted from a certain dataset may not be generalized on other datasets. For example, Shahriari et al. manually extracted the same features on two datasets: the UCSF and Cardiology Challenge 2011 database, but their model had a considerable difference of performance on another two datasets (UCSF vs. Cardiology Challenge 2011: accuracy: 93.1% vs. 82.5%, sensitivity: 96.3% vs. 83.9%, specificity: 90.0% vs. 77.7%) [12]. Manually extracted features generalized for different datasets are usually impractical in view of the costly and limited medical databases.

In this study, the proposed ECG quality assessment system consists of two stages:data augmentation using adversarial networks, and quality assessment using deep neural networks. The goal of data augmentation is to generate versatile ECG to improve the training efficiency. The proposed system can automatically extract features from raw ECG signals and make final decisions. In this case, the system can avoid relying on experience- and database-specific manual features for model development, and thresholds or rules for decision-making; therefore, they may have better generalization ability. The system demonstrates improved performance on two different datasets, and outperforms the shallow neuronal networks model and deep neural networks model without data augmentation. All the experiments were conducted using MATLAB R2019b [16] and TensorFlow 2.3.0. [17].

## 2. Materials and Methods

### 2.1. Datasets Introduction and Construction

This study uses data from PhysioNet Computing in the Cardiology Challenge 2017 (PCCC2017) database [18], TELE ECG database [19], MIT-BIH arrhythmia database (MIT-BIHA) [20], and MIT-BIH normal sinus rhythm database (MIT-BIHNSR) [21].

PCCC2017 aims to classify single-lead ECG recordings to the sinus rhythm, atrial fibrillation (AF), alternative rhythm, or as too noisy. All the recordings last for 9 to 60 s, sampled at 300 Hz. Then, each recording was resampled to 500 Hz and segmented into segments of 10 s duration with 2 s and 4 s overlap, respectively, to increase the number of unacceptable segments (in the noisy category). In total, there are 555 unacceptable and select 2618 acceptable ECG segments from this dataset, each with 10 s duration.

The TELE ECG database was initially recorded by Redmond et al. [11] from 288 home-dwelling patients. Each ECG recording was collected with single-lead and sampled at 500 Hz. Khamis et al. regarded this database as poor-quality telehealth ECG [22]. In this study, all the recordings are marked as noisy ECG signals as well. After signal segmentation, there are 734 unacceptable 10 s ECG segments.

The PCCC2017 and TELE ECG database officially provided specific quality labels for ECG segments, and then the two databases are combined to a new dataset named as COMD. COMD consists of 1289 unacceptable and 2618 acceptable ECG segments in total.

MIT-BIHA contains 48 two-channel ECG recordings, each with a duration of 30 min, sampled at 360 Hz. MIT-BIHNSR includes 18 long-term ECG recordings, and all the recordings are sampled at 128 Hz. The MIT-BIH noise stress test database (MIT-BIHNST) was created by using two clean ECG recordings (118 and 119) from the MIT-BIHA and adding noise recordings on them [7]. The noise recordings are available in MIT-BIHNST, including baseline wander (bm), muscle artifact (ma), and electrode motion artifact (em). Inspired by the construction method of MIT-BIHNST, the original ECG recordings in MIT-BIHA and MIT-BIHNSR as regarded clean signals (acceptable recordings) as well in this study, and then the same noise-added rules as MIT-BIHNST are followed to recreate a new noise-included dataset (RECD for short) using a WFDB software package [23]. The recreated ECG recordings will include severe “ma”, “bm”, or “em” noises provided by MIT-BIHNST or a Gaussian noise and power interference simulated by MATLAB. The detailed noise-added rule is listed in Table 1, where “g” means Gaussian noise generated by a MATLAB function “awgn” in the WFDB software package, and “p” means a power interference simulated by a sine function, with 60 Hz frequency. To increase data diversity, RECD is created by complying with different noise combinations. Then, all the ECG recordings in RECD are resampled at 500 Hz. After that, there were 7557 unacceptable and 20114 acceptable ECG segments available.

ECG segments in COMD were used to train a conditional generative adversarial networks (CGANs) model for ECG segment generation at first. Then, generated unacceptable and real acceptable ECG data were used to pre-train a quality assessment model. Finally, training sets in COMD and RECD were both used for fine-tuning the assessment model, and testing sets were used to test the model. The detailed usage of data is illustrated in Table 2.

### 2.2. Methods

#### 2.2.1. Data Augmentation

**Figure 1:**
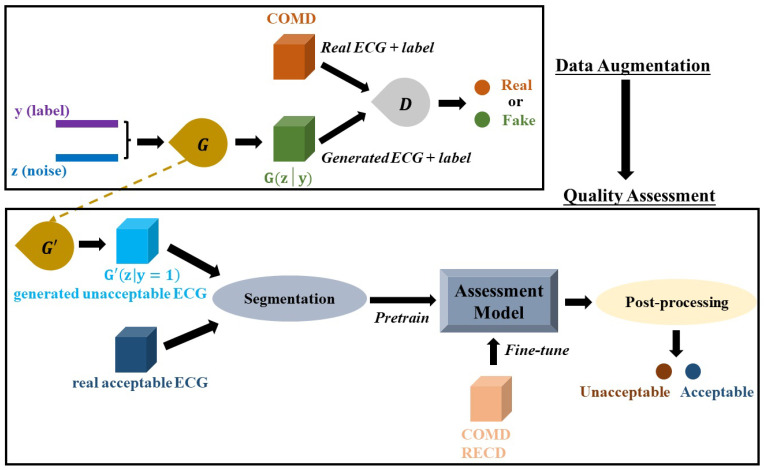
The proposed ECG quality assessment system. It consists of two parts: data augmentation by CGANs and a quality assessment model.

**Figure 2:**
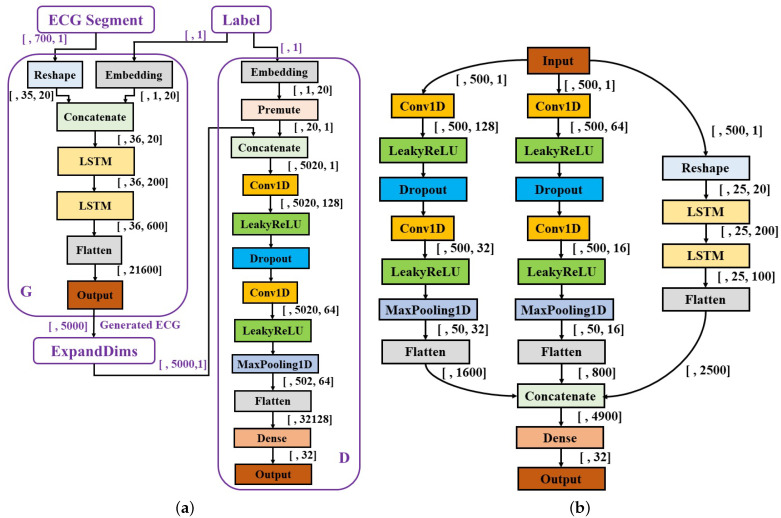
Structures of the proposed models. (**a**) The structure of CGANs; (**b**) the structure of the assessment model.

Insufficient and imbalanced data may reduce the performance of deep learning models [24,25]. Thus, the first procedure of the work was to automatically generate unacceptable ECG segments to solve data imbalance issues and perform data augmentation. Although traditional mathematical modeling methods can generate realistic heartbeats, the synthetic heartbeat’s morphology lacks diversity or is even almost the same as those of training data [26]. Recently, several studies have confirmed that the generative adversarial networks (GANs) model has the ability to generate real-like ECG segments and arrhythmia [26,27,28,29,30,31]. The proposed system is shown in Figure 1, a GANs model [32] is developed and trained based on COMD to obtain an ECG generator (G’), and G’ is used to generate unacceptable ECG segments with 10 s duration (G’(z|y = 1)). The generator and discriminator are abbreviated as G and D, respectively. Figure 2a shows the structure of the proposed CGANs model. The label information is used as the condition, and each label (“0” for acceptable and “1” for unacceptable) is assembled to an M1-element vector representation, one input of G. The other input of G is a random M2-element noise signal, and following the uniform distribution, their amplitude is limited in –1 to 1. Here, M1 and M2 are determined to be 20 and 700, respectively, by trials and errors. G mainly consists of two LSTM layers with 200 and 600 units, respectively. The main layers of D are two convolutional neural network (CNN) layers, with 128 and 64 units, respectively. Their kernel sizes are set to 10 and 5, respectively, and the alpha of the “LeakyReLU” activation layer is set to 0.2. The dropout rate is 0.3. The Dense layer has 32 units and uses “ReLu” as the activation function. For the output layer, we use “sigmoid” as its activation function. The Adam optimizer with a 0.0002 learning rate and binary crossentropy loss function are applied to train the CGANs model. By trials and errors, D is updated three times, and G is then updated once to train the model. After that, an ECG generator (G’) is obtained from the CGANs model for unacceptable ECG segment generation.

#### 2.2.2. Quality Assessment

The generated unacceptable ECG segments and parts of real acceptable segments in RECD (5000 unacceptable and 5000 acceptable ECG segments in total) were used to pre-train the quality assessment model. The structure of the model is shown in Figure 2b, and consists of three branches: two CNN branches (branch1: left; branch2: middle) and an LSTM branch (branch3: right). For branch1, the number of filters in the two CNN layers is 128 and 32 with a kernel size of 50 and 10, respectively. A dropout rate was set to 0.3, and the pooling size to 10. Branch2 has the same structure as branch1, where its two CNN layers use 64 and 16 filters and the kernel size of each is 25 and 2, respectively. The dropout rate and pooling size are the same as branch1. The number of units in the two LSTM layers of branch3 are 200 and 100, respectively. The Dense layer has 32 units with a “ReLu” activation function. A batch size of 64, an Adam optimizer with a 0.0002 learning rate, and binary crossentropy loss function were applied for training. For model fine-tuning, the three branches were frozen and only parameters in the final two layers (Dense and Output layers) were updated with real data.

**Figure 3:**
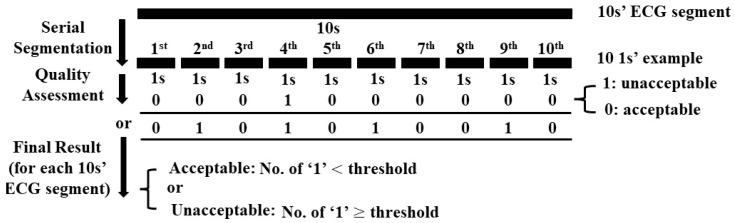
Post-processing for quality assessment of ECG segment.

Considering the limited number of data in COMD, each 10 s ECG segment was further segmented into 10 examples with 1 s duration to increase the number of input examples for the training of the assessment model. Because our aim was to assess the quality of each 10 s ECG segment, we conducted a post-processing procedure as shown in Figure 3. The threshold was set to 3 for the quality assessment of ECG segment by trial an error. After model pre-training, the COMD and RECD datasets were randomly split 10 times to obtain training sets, which were used for model fine-tuning, and testing sets, which were used for testing the average performance of the model. For fair comparison, the same number of ECG segments in COMD and RECD datasets was used for fine-tuning (3000 segments with 1000 unacceptable and 2000 acceptable), and the rest were used for testing.

## 3. Results

### 3.1. Data Augmentation

**Figure 4:**
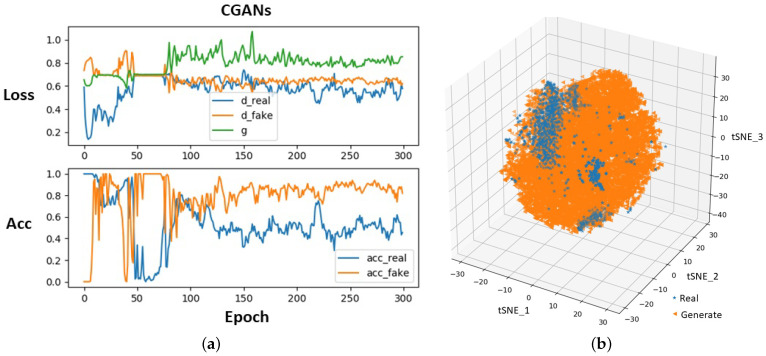
Training process of CGANs (**a**) and visualization of ECG segment distribution (**b**).

**Figure 5:**
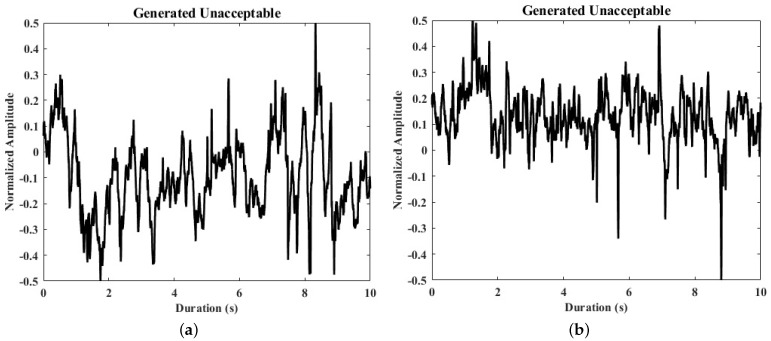

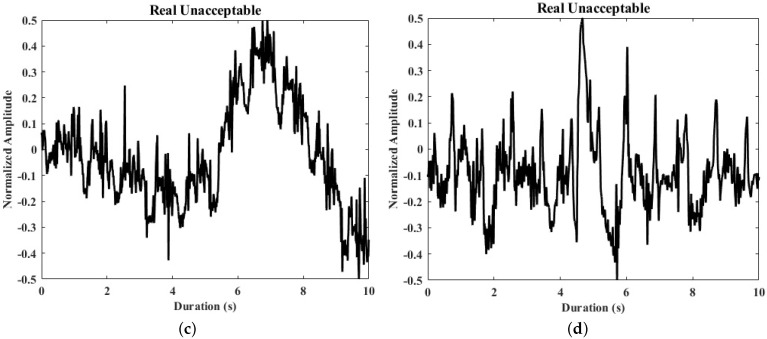
Samples of real and generated ECG segments. (**a**,**b**) The generated unacceptable ECG segments; (**c**,**d**) the real unacceptable ECG segments from the derivation dataset.

Figure 4 illustrates the training curves of CGANs and confirms their convergence. In Figure 4a, for the “Loss” figure, the blue line “d_real” means the loss of D when it is updated by real ECG segments, the orange line “d_fake” is the loss of D when it is updated by generated segments, and the green line “g” represents the loss of G. T-distributed stochastic neighbor embedding (tSNE) [33] was used to map real and generated segments to a three-dimensional space for visualization, as shown in Figure 4b. tSNE maps the similar segments to close points, and dissimilar segments were mapped to distant points. Figure 5 visualizes several real segments and generated segments by G’.

### 3.2. Quality Assessment

The performance of the proposed quality assessment model was measured by three indexes: accuracy, sensitivity, and specificity, which were calculated based on true positive (*TP*), true negative (*TN*), false positive (*FP*), and false negative (*FN*), as shown as follows.

(1) $$Accuracy=\frac{TP+TN}{TP+TN+FP+FN},$$

(2) $$Sensitivity=\frac{TP}{TP+FN},$$

(3) $$Specificity=\frac{TN}{TN+FP}.$$

**Figure 6:**
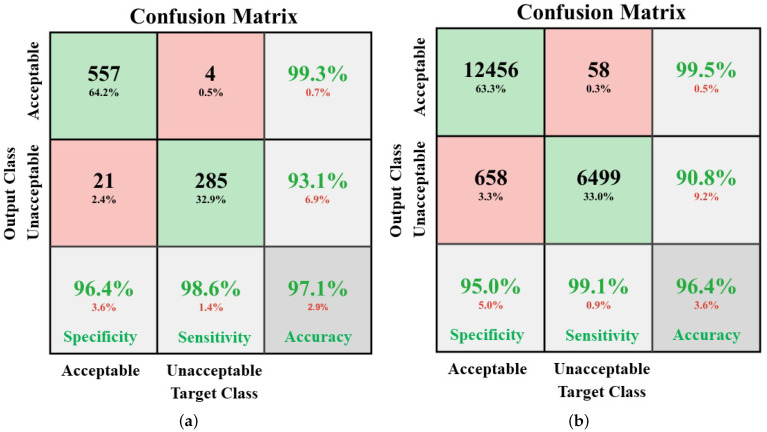
Performances of the proposed quality assessment model on COMD (**a**) and RECD (**b**).

Figure 6a,b shows the average performance of the assessment model. The specificities are 96.4% and 95.0%, sensitivities are 98.6% and 99.1%, and accuracies are 97.1% and 96.4%, respectively for COMD and RECD.

For comparison, some additional experiments were conducted as listed in Table 3. A CGANs model for 1 s example generation was developed. The quality assessment model performs 95.5% accuracy (acc), 94.5% sensitivity (sen), and 96.0% specificity (spe) on COMD, all lower than the performance of the proposed system. In addition to CGANs, a GANs model was also developed for data augmentation. The model failed in convergence when trained for 10 s ECG segments generation, but it was possible to generate 1 s ECG examples. Using the data generated by the GANs model, the quality assessment model shows an accuracy of 95.4%, sensitivity of 99.3% and specificity of 93.4% on COMD, which are not as good as the performance of the proposed method.

Moreover, to prove the necessity of training the quality assessment model using examples with a duration of 1 s, the quality assessment model was pre-trained directly using ECG segments with 10 s duration, and adding L2 regularization in CNN, LSTM, and Dense layers to alleviate overfitting; however, the model still performed differently between the training and testing sets. For COMD, the training accuracy of the model was 95.8%, the sensitivity was 91.2%, and the specificity was 98.0%, while the testing accuracy, sensitivity, and specificity were 84.4%, 75.8%, and 88.2%, respectively. This may indicate that training by segments with 1 s may improve the generalization of the quality assessment model.

Without data augmentation and pre-training the assessment model by generated ECG data, each 10 s ECG segment was segmented to 10 examples with 1 s duration to directly increase the number of examples and then use them to directly develop the assessment model. Its performance declined on both COMD and RECD compared with the proposed system, as listed in Table 3.

In addition to data generation, downsampling is a possible way to assist to train a deep learning model [34]. Thus, a comparison with a previous quality assessment method [35] was conducted. The previous study downsampled ECG segments from 500 Hz to 50 Hz and developed a shallow neural networks model to avoid overfitting. The accuracy of the proposed system increased by 1.3% and 2.6%, respectively, on COMD and RECD.

**Table 3:** Performance of models for quality assessment.

Data Augmentation		Performance of Quality Assessment		Remark
Model	Duration of Generated ECG	COMD	RECD	
CGANs	10 s	acc: 97.1%; sen: 98.6%; spe: 96.4%	acc: 96.4%; sen: 99.1%; spe: 95.0%	Proposed method
CGANs	1 s	acc: 95.5%; sen: 94.5%; spe: 96.0%	-	-
GANs	10 s	-	-	GANs: convergence failed
GANs	1 s	acc: 95.4%; sen: 99.3%; spe: 93.4%	-	-
CGANs	10 s	acc: 84.1%; sen: 75.8%; spe: 88.2%	-	Directly using 10 s ECG segments for assessment model development, and adding L2 regularization in CNN, LSTM, and Dense layers, but the model still performs overfitting; acc: 95.8% vs. 84.1% (trainng set vs. testing set); sen: 91.2% vs. 75.8%; spe: 98.0% vs. 88.2%
-	-	acc: 94.1%; sen: 96.5%; spe: 92.9%	acc: 94.0%; sen: 98.1%; spe: 91.9%	Without data augmentation, but segment each 10 s ECG segment to 10 examples with 1 s duration to naturally increase the number of examples.
-	-	acc: 95.8%; sen: 96.5%; spe: 95.5%	acc: 93.8%; sen: 89.0%; spe: 96.2%	Using shallow model and downsampled ECG segments, which is similar to the previous work [35], to avoid overfitting.

## 4. Discussion

In this study, ECG generated by CGANs benefited the ECG quality assessment task. Although downsampling is a way to assist to train deep learning models with a small training dataset, it may limit the complexity of deep neural network models and thus reduce the final performance. In this work, with the same training dataset, after pre-training with generated ECG data, the developed deep neural networks model obtained a better performance (on COMD, data augmentation by CGANs vs. downsampling: 97.1% vs. 95.8% for accuracy, 98.6% vs. 96.5% for sensitivity and 96.4% vs. 95.5% for specificity; on RECD, data augmentation by CGANs vs. downsampling: 96.4% vs. 94.0% for accuracy, 99.1% vs. 89.0% for sensitivity and 95.0% vs. 96.2% for specificity). Finally, it just needed to retrain two layers in the whole deep model; in this way, the size of the required training set may be greatly reduced compared with training from scratch. This indicates that the GANs technique may be effective to assist the training of deep neural network models for ECG-related decision-making, such as arrhythmia detection [28].

Traditional mathematical modeling methods are limited to synthesize a normal realistic heartbeat or ECG signals generally with the same morphology [36,37]. To generate ECG signals with arrhythmia, the traditional method needs to manually control position parameters of P, Q, R, S, or T waves [36] to enrich the morphology of heartbeats. On the contrary, the GANs method can instinctively generate ECG signals with a larger diversity, which better matches with real ones [26]. The ECG signal is made up of temporal sequences, and thus, generating longer durations of ECG segments is preferred. In this work, CGANs demonstrated a reliable convergence when they were trained for generating a 10 s ECG segment, but GANs failed to converge. It may be attributed to how the discriminator in CGANs is required to not only identify generated or real ECG segments, but to also provide a correct label to each real segment. This gives a stronger constraint for model training than GANs.

In this study, the proposed system assessed the quality of each 10 s ECG segment by consecutively analyzing the quality of 10 examples, each 1 s in length. This improved the reliability of the system. For example, for a 10 s ECG segment, a threshold of 3 was set in post-processing, as shown in Figure 3; that is, if there were more than 3 out of the 10 consecutive 1 s examples which were determined as “unacceptable” by the model, the corresponding 10 s ECG segment was classified as “unacceptable”. In this case, despite the quality of the rest of the seven consecutive examples, the final result did not change and the sensitivity was assured. This characteristic is confirmed in the results, as shown in Figure 6; that is, the sensitivity of the model validated by COMD and RECD is higher than both the specificity and accuracy.

This work has some limitations, as follows.

**Figure 7:**
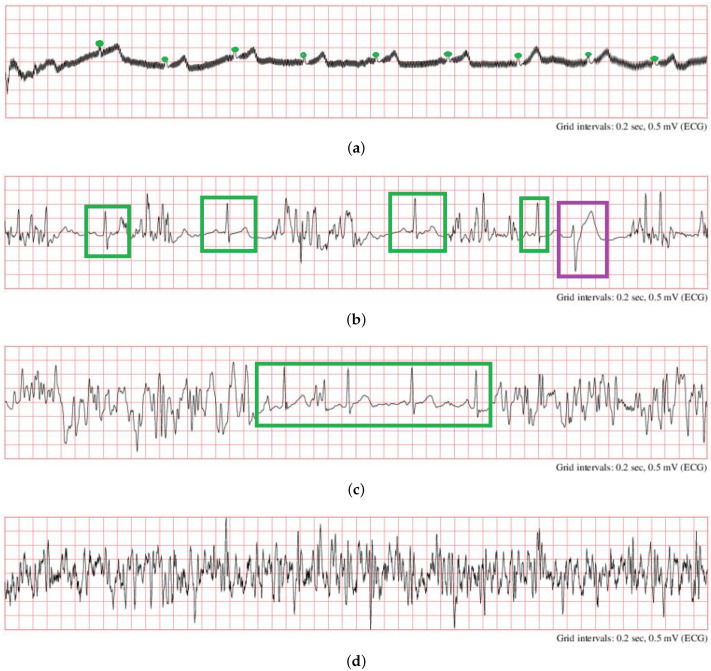
Samples of noisy ECG signals in PCCC2017 (**a**): recording “A07/A07983”, time: 10:10:15–10:10:25; (**b**): recording “A01/A01938”, time: 05:05:40–05:05:50; (**c**) recording “A00/A00445”, time: 09:09:09–09:09:19, (**d**) recording “A01/A01116”, time: 04:02:47–04:02:57 [18].

(1) The proposed system is suitable for an initial ECG quality assessment, without considering specific applications. The quality requirement may vary for different purposes. For better explanation, several ECG segments that were labeled as “noisy” in PCCC2017 are shown in Figure 7. For example, for a task of AF detection, one of the characteristics for the detection of AF is that there are irregular heartbeats and no regular P waves in ECG. However, P waves in Figure 7a,b (Figure 7a: recording “A07/A07983”, time: 10:10:15–10:10:25; Figure 7b: recording “A01/A01938”, time: 05:05:40–05:05:50) were hardly observed because of severe noise; thus, the quality of ECG segments in Figure 7a,b is unacceptable because they cannot be used for further AF detection using P waves. In contrast, the segment in Figure 7a can be used for HRV time-domain analysis since it has obvious R peaks (green circle), and thus, the heart rate can be accurately calculated. In this case, it should be regarded as acceptable. Moreover, it can identify premature ventricular contraction (PVC) rhythms in Figure 7b (purple rectangle) and normal beats (green rectangles); thus, it is acceptable when used for PVC detection. In Figure 7c (recording “A00/A00445”, time: 09:09:09–09:09:19), the ECG segment can be partly regarded as acceptable (green rectangle) or unacceptable (two sides). The ECG segment in Figure 7d (recording “A01/A01116”, time: 04:02:47–04:02:57) should be totally unacceptable because the signal is completely contaminated by noise.

(2) In this study, the system only considered a quality assessment for single-lead ECG; therefore, the proposed method cannot be directly applied to 12-lead ECG quality assessments. The system is suitable for the quality assessment of ECGs collected by bedside monitors or wearable devices, but should be improved for the diagnosis of cardiovascular diseases using 12-lead ECG, such as acute myocardial infarction.

(3) In this study, the proposed system was unable to provide specific signal-to-noise ratio information for acceptable and unacceptable ECG signals; thus, it may be hard to quantize the quality of ECG signals.

In future, it is expected that we develop a multi-hierarchical and meticulous ECG quality assessment system. The system will identify low-quality ECG signals and perform task-specific quality assessments.

## 5. Conclusions

The CGANs technique is a possible method for ECG generation, and the generated data will help to improve the results of ECG quality assessments. The proposed system is expected to be applied for the accurate initial screening of ECG quality. In particular, for patients with wearable ECG recording devices, the system may assist inexperienced users to collect ECG signals with a quality that meets diagnostic requirements. For clinical technicians, the proposed system is expected to relieve them from tedious and time-consuming quality screening work.

## Figures and Tables

**Table 1:** The noise-add rules of dataset recreation.

Noise Type	MIT-BIHA	MIT-BIHNSR
bw	-	“19093”, “19140”, “19830”
em	-	All 17 recordings
ma	“101_V1”, “106_V1”, “112_V1”, “113_V1”, “114_V5”, “115_V1”, “122_V1”, “200_V1”, “205_V1”, “209_V1”, “215_V1”, “220_V1”, “221_V1”, “222_MLII”	All 17 recordings
bw, g	-	Recordings expect “19093”, “19140”, “19830” (Total 14 recordings)
bw, p	-	Recordings expect “19093”, “19140”, “19830” (Total 14 recordings)
em, g	“101_V1”, “106_V1”, “112_V1”, “113_V1”, “114_V5”, “115_V1”, “122_V1”, “200_V1”, “205_V1”, “209_V1”, “215_V1”, “220_V1”, “221_V1”, “222_MLII”	-
ma, bw	“112”, “113”, “114”, “115”, “116”, “117”, “118”, “119”, “121”, “122”, “123”	-
ma, em	“124”, “200”, “201”, “202”, “203”, “205”, “207”, “208”, “209”, “210”, “213”, “214”, “215”	All 17 recordings
bw, g, p	“101_V1”, “106_V1”, “112_V1”, “113_V1”, “114_V5”, “115_V1”, “122_V1”, “200_V1”, “205_V1”, “209_V1”, “215_V1”, “220_V1”, “221_V1”, “222_MLII”	-
em, bw, g	“212”, “217”, “219”, “220”, “221”, “228”, “230”, “231”, “232”, “233”, “234”	-
ma, em, bw	“100”, “101”, “102”, “103”, “104”, “105”, “106”, “107”, “108”, “109”, “111”	-
g	first 5 min of each recording	first 5 min of each recording
p	first 5 min of each recording	first 5 min of each recording

**Table 2:** Usage of datasets.

Usage	COMD		Generated Unacceptable ECG	RECD		
	Training Set	Testing Set		Parts of Acceptable ECG	Training Set	Testing Set
Train CGANs	*√*		-	-		
Pretrain Assessment Model	-		*√*	*√*	-	
Finetune Assessment Model	*√*	-	-	-	*√*	-
Test Assessment Model	-	*√*	-	-	-	*√*

## Data Availability

(1) The PhysioNet Computing in Cardiology Challenge 2017: https://physionet.org/content/challenge-2017/1.0.0/ (accessed on 1 December 2020); (2) TELE ECG Database: https://dataverse.harvard.edu/dataset.xhtml?persistentId=doi:10.7910/DVN/QTG0EP (accessed on 1 December 2020); (3) MIT-BIH arrhythmia database: https://physionet.org/content/mitdb/1.0.0/ (accessed on 1 December 2020); (4) MIT-BIH Normal Sinus Rhythm Database: https://www.physionet.org/content/nsrdb/1.0.0/ (accessed on 1 December 2020).
